# The potential impact data of Tylosin and Enrofloxacin veterinary antibiotics on germination and accumulation in barley seed as a forage crop and good dietary sources using LC/MS-MS

**DOI:** 10.1016/j.dib.2019.104326

**Published:** 2019-07-27

**Authors:** Ahmed Abou Elezz, Ahmed Easa, Fathy Atia, Talaat Ahmed

**Affiliations:** a Environmental Science Center (ESC), Qatar University, P.O. Box 2713, Doha, Qatar; b Central Laboratory Unit, Qatar University, P.O. Box 2713, Doha, Qatar

**Keywords:** Phytotoxic effect, Veterinary antibiotics, Seed germination, Barley, Spectrometry

## Abstract

In this study, the phytotoxic effects caused by the exposure to five different concentrations of two veterinary antibiotics (Tylosin, and Enrofloxacin) that are commonly used for the treatment of farm animals as antibacterial agents were considered. The impact of antibiotic residues was evaluated on the germination percentage, accumulation, and seedling elongation of the barley seeds using Petri dishes under controlled environmental conditions. The treatments were distributed randomly using Completely Randomized Design (CRD). The germination percentage was significantly inhibited with the increasing Enrofloxacin dose concentrations, while, it was to some extent on the contrary in the case of Tylosin, where seed germination was enhanced as a result of increasing Tylosin concentrations.

Liquid chromatography-tandem mass spectrometry LC/MS-MS was used to detect and quantify the uptake dosage after drying and extracting the antibiotic compounds from the seedling.

**Table:** Specifications Table

Subject	Agricultural and Chemical Sciences
Specific subject area	Phytotoxic effect on seed germination
Type of data	Table, figure, chart
How data were acquired	Liquid chromatography (LC/MS-MS) was used to obtain the impact of veterinary antibiotics on seed germination, and data was investigated using SPSS Package 25 and MS Excel 2016.
Data format	Raw, analyzed
Parameters for data collection	Seed germination percentage, germination frequency, root and shoot length, seedling fresh and dry weight of barley were tested under different concentrations of two veterinary antibiotics. The uptake concentrations of two veterinary antibiotics were quantified using Liquid chromatography (LC/MS-MS).
Description of data collection	A local variety of barley Seeds were tested for germination with different concentrations (Control, 0.1, 10, 100, 500, 1000 mg. L^−1^) of Tylosin and Enrofloxacin veterinary antibiotics; then seedlings were dried and tested to quantify the accumulation of the antibiotic by LC/MS-MS.
Data source location	Institution: Environmental Science Center (ESC), Qatar University City: Doha Country: Qatar Latitude and longitude: (25°22′28.56″N, 51°29′24.72″E)
Data accessibility	The raw data was archived in Mendeley Data https://data.mendeley.com/datasets Reserved https://doi.org/10.17632/wnpk4dgpdy.2 Link to the preview: https://data.mendeley.com/datasets/wnpk4dgpdy/2

**Table:** 

**Value of the data** • LC/MS-MS method permits to extract and analyze a wide range of veterinary antibiotics concentrations (Control, 0.1, 10, 100, 500, 1000 ppm) that were accumulated through seed germination. • This study was useful to understand the potential impact of some veterinary antibiotics (Tylosin, and Enrofloxacin) on the seed germination of the barley forage crop. • Furthermore, the dataset introduced in this article can be used to investigate the impact of veterinary medical waste on seed germination percentage, root and shoot length, roots, and shoots over control percent, fresh weight, and dry weight of seedling.

## 1 Data

**Fig. 1:**
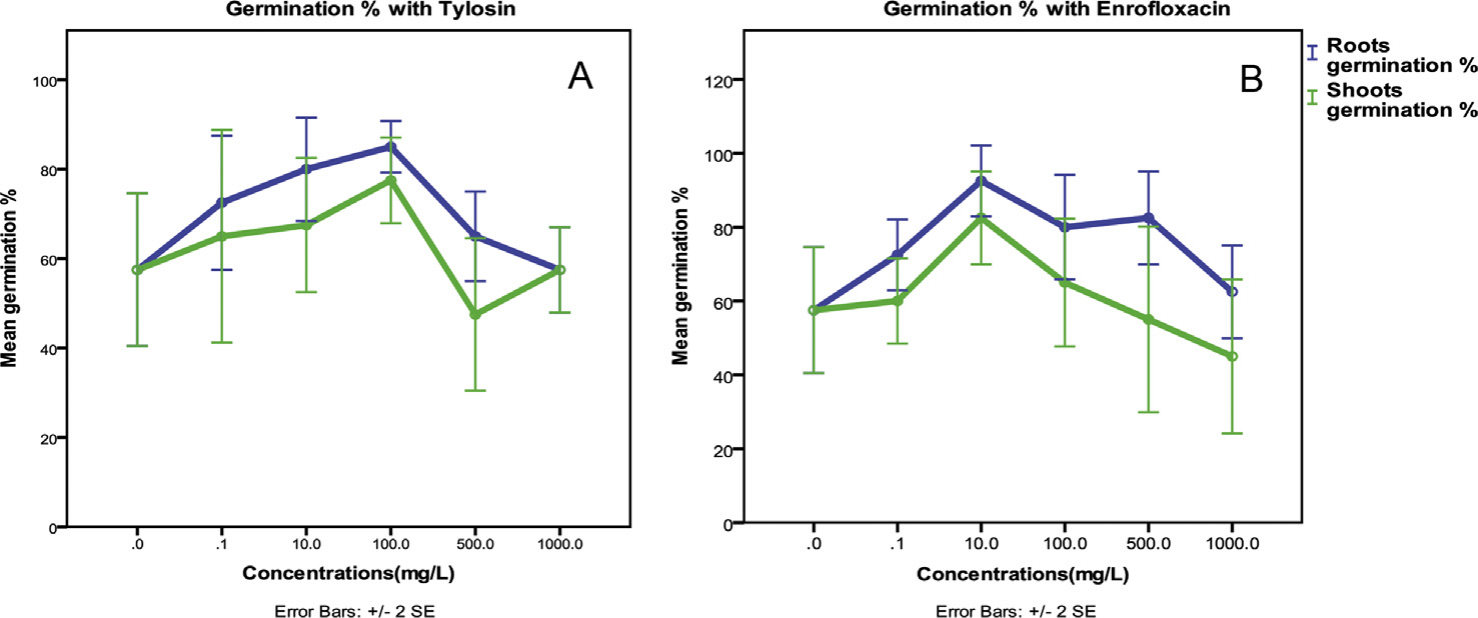
Germination percent of the shoots and roots under the effect of Tylosin (A), Enrofloxacin (B) antibiotics concentrations.

**Fig. 2:**
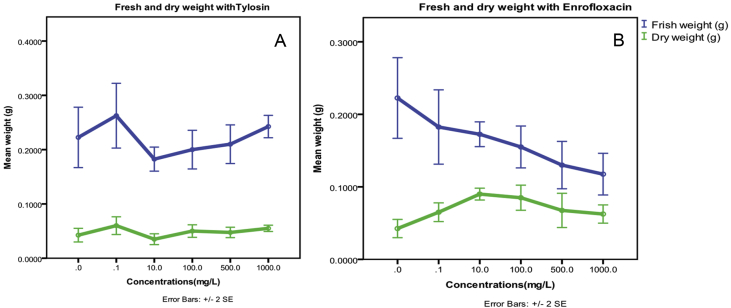
The seedling fresh and dry weight with different Tylosin (A) and Enrofloxacin (B) antibiotic concentrations.

**Fig. 3:**
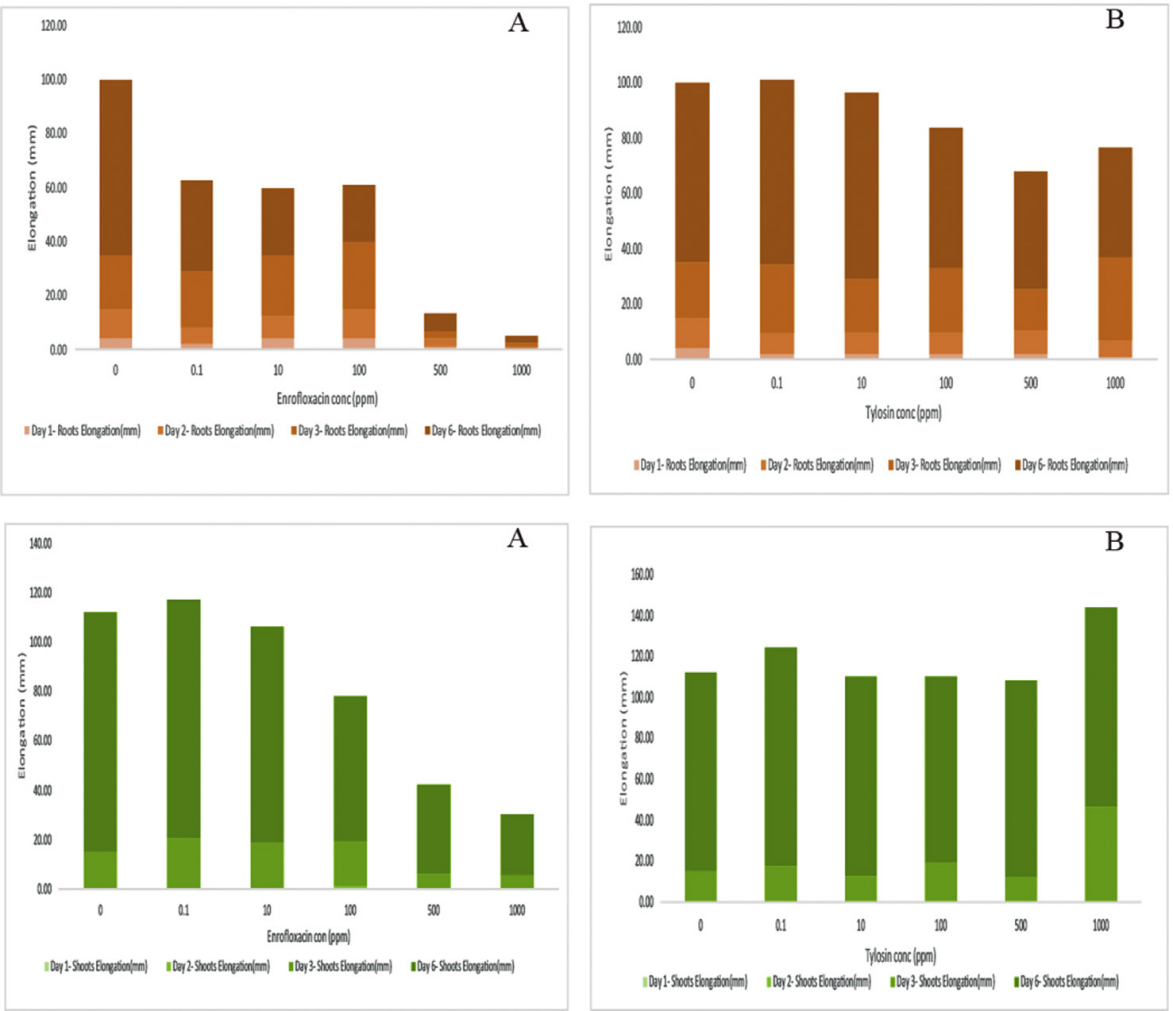
Root and shoot elongation chart with Enrofloxacin (A) and Tylosin (B) antibiotics.

Development of roots was started on day two of the seed germination without any shoots. After three days, the primary shoots (plumules) were started. Maximum elongation was found on day six of germination. The significant observation during the experiment was increasing the inhibition rate gradually with increasing the concentration of Enrofloxacin antibiotic from the control sample (0 ppm) to 1000 mg. L^−1^ (Table 1), while Tylosin was somewhat on the contrary (Fig. 1, Fig. 2, Fig. 3).

**Table 1:** Number of seeds germinated (out of 10 seeds), max elongation(mm), Roots and shoots over control %, fresh weight, dry weight of barley seed(g) and germination percentage.

Treatment	Conc mg. L^−1^	Percent of germinated seeds (out of ten)	Maximum elongation on day six		Roots over control %	Shoots over control %	Fresh weight (g)	Dry weight (g)	Root germination %	Shoot germination %
			Mean Roots Elongation(mm)	Mean Shoots Elongation(mm)						
Control	0	60%	65.0	97.4	100	100	0.22	0.04	58%	58%
Antibiotic-1 (Tylosin)	0.1	70%	66.8	106.8	103	110	0.22	0.06	73%	65%
	10	70%	67.5	97.8	104	100	0.15	0.04	80%	68%
	100	80%	50.9	91.0	78	93	0.19	0.05	85%	78%
	500	60%	42.4	96.1	65	99	0.15	0.05	65%	48%
	1000	60%	39.5	97.5	61	100	0.24	0.05	58%	58%
Antibiotic-2 (Enrofloxacin)	0.1	70%	33.9	96.9	52	99	0.15	0.06	73%	60%
	10	90%	24.8	87.6	38	90	0.16	0.09	93%	83%
	100	70%	21.3	58.8	33	60	0.13	0.09	80%	65%
	500	70%	6.60	36.0	10	37	0.08	0.06	83%	55%
	1000	50%	2.60	25.0	4	26	0.08	0.06	63%	45%

Barley seedlings elongation under the influence of different concentration levels was outlined in the bar chart (Fig. 3). Roots and shoots were gradually inhabited with increasing the Enrofloxacin concentration, especially under 500, 1000 mg/L, while it was clear that Tylosin had no significant effect.

The percent over control of roots and shoots were calculated to reaffirm the potential impact of Enrofloxacin antibiotic on the germination of barley (Table 1).

**Fig. 4:**
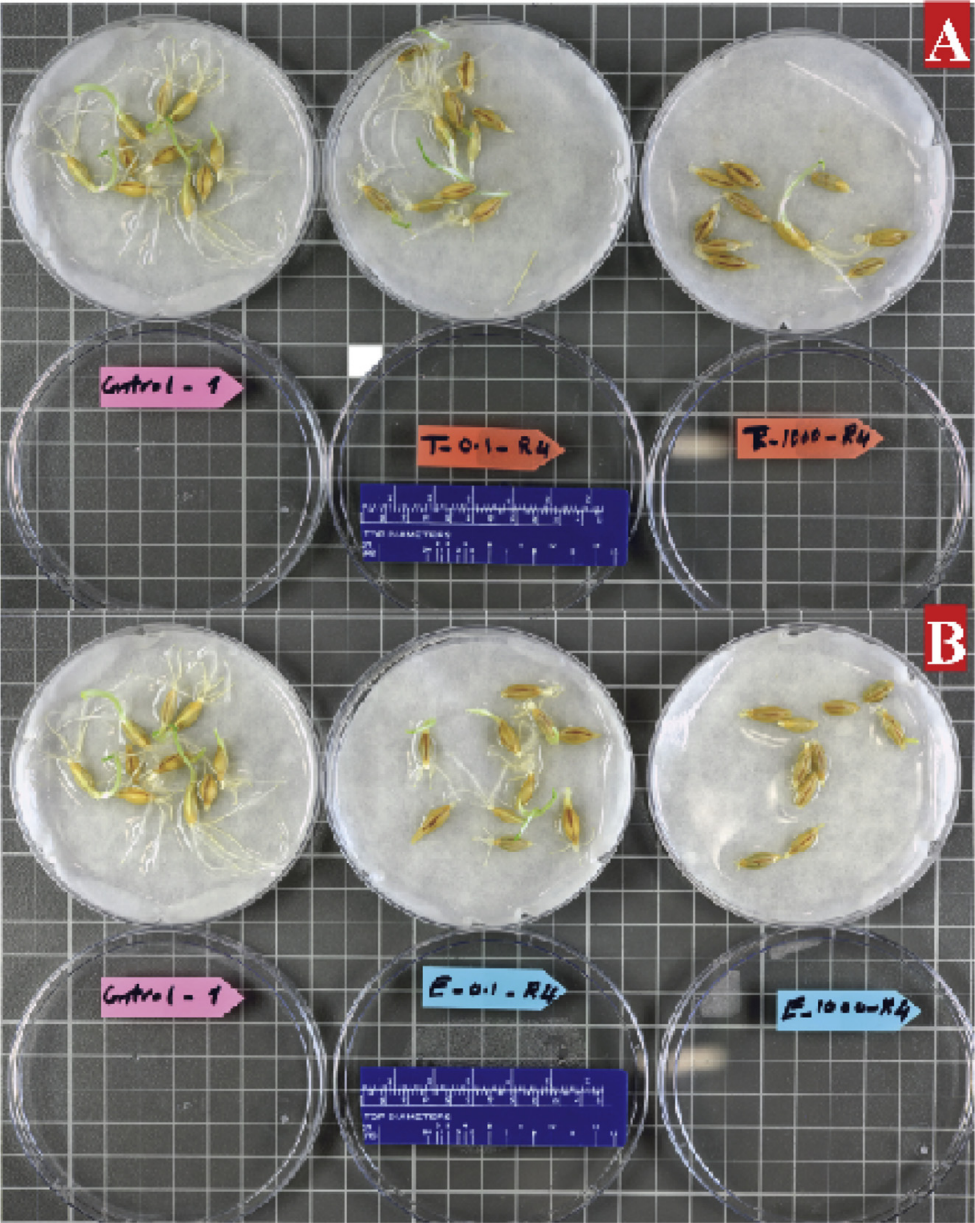
The variation between a control sample (0), 0.1, and 1000 mg/L in 2 different veterinary antibiotics on germination of barley seed. (A) Tylosin and (B) Enrofloxacin in day 4.

In Fig. 4, the variation of root and shoots germination was illustrated between the control sample and two treatment concentrations (0.1, 1000 mg/L) of Tylosin and Enrofloxacin in day 4. The variation was significant between control and 1000 mg/L in Enrofloxacin.

**Fig. 5:**
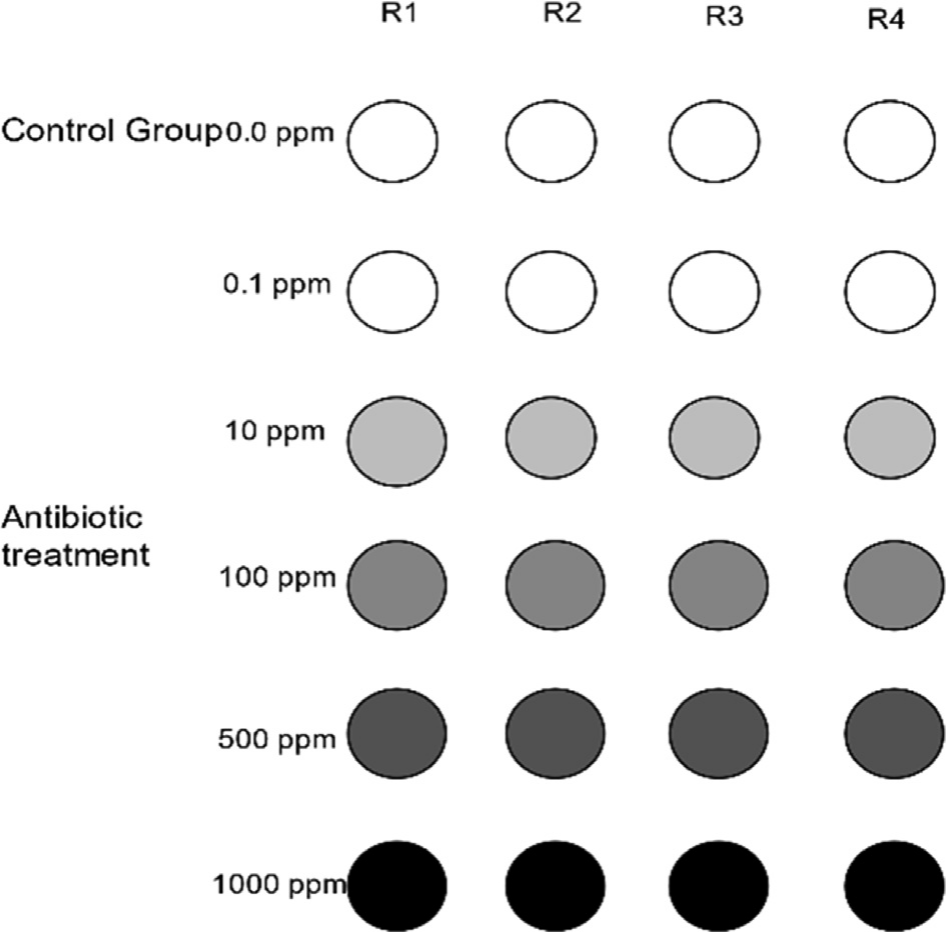
Outline of the experiment using Complete Randomized Design (CRD) with five treatments and a control sample with four replicates (R).

The Completely Randomized Design (CRD) of this study experiment was explained graphically using five treatments of Tylosin and Enrofloxacin with four replicates for each treatment (Fig. 5).

The mean uptake concentrations of four replicates (n = 4) of Tylosin and Enrofloxacin with different treatments were represented in the roots, and shoots of the barley seedlings (Table 2). The antibiotic concentrations were separated from the sample matrix using High Performance Liquid Chromatography (HPLC) system.

**Table 2:** The antibiotics uptake concentrations (n = 4) in (mg. L^−1^) of seedling under the effect of Tylosin, Enrofloxacin with different concentrations analyzed by LC/MS-MS.

Initial antibiotics concentrations (mg. L-1)	Tylosin (mg. L^−1^)		Enrofloxacin (mg. L^−1^)	
	Root	Shoot	Root	Shoot
0	0.00 ± 0.00	0.00 ± 0.00	0.00 ± 0.00	0.00 ± 0.00
0.1	0.00 ± 0.00	0.00 ± 0.00	0.01 ± 0.01	0.00 ± 0.00
10	0.00 ± 0.01	0.01 ± 0.01	0.01 ± 0.02	0.01 ± 0.01
100	0.67 ± 0.50	0.84 ± 0.81	2.23 ± 0.78	1.26 ± 0.65
500	3.65 ± 2.11	2.87 ± 1.83	10.66 ± 6.39	2.40 ± 1.64
1000	4.69 ± 1.61	3.20 ± 1.52	15.70 ± 7.56	1.84 ± 0.82

Table 4 demonstrates the characteristics of a Multi reaction monitor (MRM) method used to quantify and detect residual antibiotics (mg. L^−1^).

The data were analyzed using the analysis of variance (one-way ANOVA) under completely randomized design, and the treatment means were compared by Tukey test using IBM-SPSS statistics software (version 25).

The multiple Comparisons (Tukey HSD) test for the roots germinated % represents a significant variation (P < 0.05 at 95% confidence interval) only between control (0 mg/L) and Enrofloxacin treatments, while, the shoots germination % had no significant variation between treatment groups at the same conditions (P > 0.05 at 95% confidence interval).

A significant variation was found in the uptake concentrations between treatment groups (P < 0.05 at 95% confidence interval) in Tylosin and Enrofloxacin antibiotics (Table 3).

**Table 3:** One way ANOVA test of the antibiotics uptake concentrations between treatments.

ANOVA		df	Mean Square	F	Sig(*P*)
uptake concentration of Tylosin	Between Groups (Treatments)	4	48.300	31.140	.000
	Within Groups	12	1.551		
	Total	16			
uptake concentration of Enrofloxacin	Between Groups (Treatments)	4	188.447	7.910	.004
	Within Groups	10	23.825		
	Total	14

**Table 4:** The MRM method used to quantify the residues of veterinary antibiotics in water matrices.

Compound name	Precursor ion	Fragmentor (V)	Product-Ion	Collision energy (eV)
Tylosin	916.3	220	174	40
			101	52
Enrofloxacin	360.2	115	342.2	16
			316.2	16

## 2 Experimental design, materials, and methods

• Experimental Design

The experiment was conducted using a common forage crop plant (Barley) and included two antibiotics (Tylosin, Enrofloxacin) with five different concentrations (0.1, 10, 100, 500, 1000 mg. L^−1^) in addition to the control, and each treatment was replicated four times. It was carried out on Petri dishes under a controlled environment (Temperature and light duration). The treatments were distributed randomly using Completely Randomized Design (CRD).

Local barley Seeds were used in this study. Seeds were germinated using distilled water mixed with the antibiotics. Antibiotics were used with different concentrations ranged from 0.1 to 1000 mg. L^−1^.

Each dish accurately contains ten selected seeds by adding approximately fifteen ml of different treatments. The germination percentage (%), root length, shoot length, the fresh and dry weight of seedling were measured. Seeds were considered germination with the emergence of radical. The germination percentage was determined by counting the number of seeds germinated during the experiment period (six days) over the total number of seeds according to Equation (1), [1].

Equation 1 $$\;\text{Germination\%}=\frac{\text{Number}\;\text{of}\;\text{germinated}\;\text{seeds}}{\text{Total}\;\text{number}\;\text{of}\;\text{seeds}}\times 100$$

Fig. 5 outlines the experimental design for a forage crop seed germination under five antibiotic concentrations. Two types of antibiotics were used in this study. Each treatment was replicated four times in four Petri dishes. In total, 48 Petri dishes were used and arranged completely random under controlled condition.

The uptake and accumulation of antibiotics in the barley seedling samples were measured -after drying the samples using a freeze dryer-on the seedlings using ultrasound extraction (USE) and LC/MS-MS (6460 Triple Quad LC/MS, Agilent Technologies).

**Table:** 

Chemicals
Tylosin tartrate 20%, and Enrofloxacin 10% (Dutch Farm International, Holland)
Formic acid (reagent grade, ≥ 95%, Merck)
Methanol (HPLC grade, ≥ 99.9%, Sigma-Aldrich)
Distilled water
Preparation of standards and samples
Freeze-Dried and homogenized samples were weighed in 50 ml centrifugal tube. 10 ml of 1 mM formic acid/methanol solution (1:1) was added to each sample as an extracting solution [2].
The samples were sonicated for 2 h to extract the uptake antibiotic compounds before analysis.
The separation technique was done by using an Agilent 1290 (HPLC) system, which consists of a binary pump, autosampler, and thermostatic column oven. The separating column used was Zorbax C18 (150 mm × 2.1 mm i. d., 3.5 μm). The column temperature maintained at 30 °C. The mobile phase consists of eluent (A) 0.1% formic acid solution and eluent (B) acetonitrile.
The mass detection was done by using Agilent 6460 triple quadrupole mass spectrometer (MS/MS) with electrospray ionization (ESI) in positive ionization mode. The mass analysis was performed using (MRM), as shown in Table 4. The operation condition was as follows:
gas flow 10 L. min^−1^ at temperature 350 °C, nebulizer 30psi, sheath gas flow rate 11 L. min^−1^ at temperature 350 °C, capillary voltage 3.5kV and dwell time of 50 ms per ion pair.
